# Reviewed article: Carvalho JB, Salviano AF, Lourenço BH, Tomita LY. Temporal trend of food consumption indicators of adolescents monitored by Primary Health Care services of the Brazilian National Health System, 2015-2019. Epidemiol Serv Saude. 2025:34;e20240893

**DOI:** 10.1590/S2237-96222025v34e20240893.c

**Published:** 2025-08-04

**Authors:** Iolanda Karla Santana dos Santos

**Affiliations:** Universidade Federal do ABC

**Table:** 

Rating	Excellent	Good	Average	Below average	Poor
Interest		✓			
Quality		✓			
Originality		✓			
Overall		✓			

**Table:** 

Questionnaire	Yes	No	Not applicable
Does the manuscript contain new and significant information to justify publication?	✓		
Does the Abstract (Summary) clearly and accurately describe the content of the article?	✓		
Is the problem significant and concisely stated?	✓		
Are the methods described comprehensively?	✓		
Are the interpretations and conclusions justified by the results?	✓		
Is adequate reference made to other work in the field?	✓		

## Manuscript Structure

Length of article is: Adequate

Number of tables is: Adequate

Number of figures is: Adequate

Please state any conflict(s) of interest that you have in relation to the review of this paper (state “none” if this is not applicable): None

## Comments

Inicialmente, parabenizo a iniciativa dos/as autores/as em estudar os indicadores do consumo alimentar no grupo de adolescentes, o qual por vezes é esquecido.

Trata-se de estudo de séries temporais com o objetivo de avaliar a tendência temporal de práticas alimentares de adolescentes acompanhados na Atenção Primária à Saúde, 2015-2019. Os/as autores/as observaram aumento da prevalência de indicadores da alimentação saudável e também do consumo de alimentos ultraprocessados.

A pesquisa contribui para o SUS na medida em que utiliza dados produzidos na rotina dos cuidados em saúde da APS.

Segue abaixo algumas sugestões e dúvidas que emergiram durante a leitura do manuscrito:

Página 05: Na construção dos indicadores, a resposta ao questionário com “não sabe” foi contabilizada no denominador?

Página 05: Na análise de dados, na fórmula, acho que o ponto de min. e máx. não está sobrescrito. Nas tabelas, a formatação está diferente.

No título da Tabela 1 e da Tabela 2, a letra “e” está duplicada. Além disso, está somente segundo sexo, e nas tabelas são apresentadas outras estratificações (faixa etária e macrorregião).

Página 08: Seria interessante atualizar o trecho sobre a cesta básica que foi votada em dezembro/2024.

